# Practical Notes and Formulæ

**Published:** 1882-01-20

**Authors:** 


### PRACTICAL NOTES AND FORMULÆ.

**Absorbent Cotton.**—The American Journal of Pharmacy says: Take of the best quality of carded cotton batting, any desired quantity, and boil it with a 5 per cent. solution of caustic potassa or soda for one-half hour, or until the cotton is entirely saturated with the solution, and the alkali has saponified all oily matter. Then wash thoroughly, to remove all soap, and nearly all alkali; press out the excess of water, and immerse in a 5 per cent. solution of chlorinated lime for 15 or 20 minutes; again wash, first with a little water, then dip in water acidulated with hydrochloric acid, and thoroughly wash with water; press out the excess of water, and again boil for 15 or 20 minutes in a 5 per cent solution of caustic potass; now wash well, dipping in the acidulated water and washing thoroughly with pure water. Afterwards press out and dry quickly.—[Ex.

**Pruritus Vulvæ.**—In the acute stage of pruritus accompanying vulvitis, emollient applications are, of course, indicated. Starch poultices (not linseed, for this decomposes too easily), lotions of infusion of belladonna, aconite, or poppy-heads, or of a weak solution of bromide of potassium or of chloral (three grains to the ounce), may be used. They should be hot rather than cold. Washes of corrosive sublimate of one-per-cent. strength may be employed when the stage of acute inflammation is passed.

Fifty parts perfectly neutral glycerole of starch, containing one part of the following substances, tannin, calomel, extract of belladonna, or oil of cade, according to circumstances, may be used with advantage. Now and then light cauterizations with nitrate of silver prove advantageous. Revillout has occasionally found that the insertion of slices of citron between the vulva will allay the itching. In chronic cases Dr. Gueneau de Mussy anoints the vulva night and morning with the following—

| | | |
|---|---|---|
| ℞ | Glycerol. amyli, ............................ | ℥j, |
| | Potassii bromidi........................ | aa gr. xxv, |
| | Bismuthi subnit........................ | |
| | Hydrarg. chlor. mite........................ | gr. x, |
| | Ext. belladonnæ.......................... | gr. v. |

M. The vulvæ are to be washed with a dilute solution of borax containing a little emollient, as starch.

Delioux de Savignac follows the lotion just mentioned with a powder:

| | | |
|---|---|---|
| ℞ | Pulv. lycopodii.................................. | ℥j, |
| | " bismuthi subnit........................ | ʒiss, |
| | " radicis belladonnæ..................... | ʒss. |

M. In very rebellious cases, hip-baths, each containing two to three drachms of corrosive sublimate, first dissolved in dilute alcohol, may be employed.—Med. Times.

**For Itching.**—The late Dr. Tilbury Fox, (in Practitioner) insisted particularly on the use of soothing remedies in various forms of skin disease where there was irritation to be allayed. These may consist of substances in the state of powder, as the carbonate and oxide of zinc, bismuth, chalk, etc., suspended in water, to which a little glycerine has been added. Glycerine when so used must be the best and purest, free from fatty acids, and must be well diluted. Pure glycerine undiluted is an irritant, freely diluted with water is an emollient, and the best remedy we have for that dry and fissured state of the cuticle occurring in the hands and face and legs during frost or when east winds are prevalent. The same softening effect is produced when glycerine and starch are combined in the glycerinum amyli of the Pharmacopœia, the starch here serving both to dilute the glycerine and also to impart its own demulcent properties.—[Monthly Review of Med. and Pharmacy.

**Injection Brou.**—The following is given as formula for this famed gonorrhœa injection: sulphate of zinc, eight grams; acetate of lead, fifteen grains; tincture of catechu, two drachms; aqeous tincture of opium, three ounces. The formula of the aqueous tincture of opium is known to but few pharmacists, and it is, therefore, not easily obtained. It is, however, not impossible that a dilution of tincture of opium would answer all purposes.—[Monthly Review.

**The Bromides.**—Dr. Sequin thus prescribes the bromides—

℞ Potassii bromidi..............................℥jss.

Aquæ,..................................f℥vij.

A teaspoonful contains fifteen grains of the salt.

Another formula which he often employs is:

℞ Ammonii bromidi........................℥ss.
Potassii bromidi........................℥j.
Aquæ...............................f℥vij. M.

Of this solution also a teaspoonful contains fifteen grains of the salts.—[Medical Times.

**Formulæ Used in the New York Hospital—For external use.**

ANTISEPTIC SOLUTIONS.

Sol. acid carbolic..........................1-20 water.
Sol. acid carbolic ..........................1-30 water.
Sol. acid carbolic ..........................1-40 water.
Sol. acid boracic ..........................1-30
Sol. thymol..............................1-1000

Distilled water is preferred; it will make a clearer solution than ordinary water.

WARD GARGLE.

℞ Tannin.............................. ʒ ss

Sol. potassii chlorat. sat.................. ℥ viij

M.

### CARBOLIC SPRAY.

℞ Sodii bicarb.,<br>

Sodii biborat............................aa ʒj<br>

Acidi carbolici......................... gr. xl<br>

Glycerine............................... ʒvij<br>

Aquæ................ ................ad ℥viij<br>

M.

### MURIATE OF AMMONIA WASH.

℞ Ammonii chloridi........................ ... ℥ ss<br>

Tinct. opii.............................ad ℥ j<br>

Aquæ.................................... Oij<br>

M.

### CHURCHILL'S TINCTURE OF IODINE.

℞ Iodinii.................................. ʒ j<br>

Potassii iodidi.......................... ʒ ij<br>

Aquæ destill.,<br>

Alcohol..............................aa f. ℥ ij

### PARASITICIDE.

℞ Acidi carbol..... ........................ grs. x<br>

Ungi. hydrarg nitrat.,<br>

Sulphur. precip.......................aa ʒ j<br>

Ungt. simplicis.......................... ℥ j

### STIMULATING LOTION.

℞ Arnicæ tinct............................ *m* xx<br>

Spts. rosmarin........................... *m* xv<br>

Aq. dest................................. ℥ j

### CARBOLIZED VASELINE.

℞ Vacelinæ................................ ℥ xx<br>

Acid. carbolic. crystal.................. ... ℥ j<br>

Melt each separately and mix.

### OINTMENT OF CHRYSOPANIC ACID CONCENTRATED.

℞ Acid. chrysophanic.......... ........... ... ℥ j<br>

Ung. simplicis........................... ℥ iv<br>

Melt the ointment, and while hot add the acid, stirring till dissolved.

### OINTMENT OF SALICYLIC ACID.

℞ Pulv. acid. salycilic...................... ʒ j<br>

Vaselinæ................................. ℥ j

### CHLORATE OF POTASSA MIPTURE.

℞ Ammon. muriat.,<br>

Potass. chlorat........................aa ʒ j<br>

Ext. glycyrrh............................ ʒ ss<br>

Aquæ cinnam............. ...........ad ℥ iv<br>

Dose, a tablespoonful.

